# Supplementary material for: Retrosplenial cortex enables context-dependent goal-directed sensorimotor transformation
Source: eLife. 2026 Jun 29;14:RP109717. doi: 10.7554/eLife.109717 (PMC13313688; doi:10.7554/eLife.109717)
Supplement: Peer review history

# Editor report: eLife Assessment

**DOI:** 10.7554/eLife.109717.3.sa0

**Authors:** Leopoldo Petreanu

**Affiliations:** Champalimaud Center for the Unknown Portugal

**Keywords:** Compelling, Important

This **important** study combines optogenetic manipulations with widefield cortical imaging to investigate the neural basis of context-dependent sensory processing. It provides **compelling** evidence that the retrosplenial cortex modulates behavioral responses to whisker deflection depending on the behavioral context. The paper will be of strong interest to neuroscientists studying cortical mechanisms of sensorimotor processing.

# Referee report: Reviewer #1 (Public review)

**DOI:** 10.7554/eLife.109717.3.sa1

Summary

The strength of this manuscript lies in the behavior: mice use a continuous auditory background (pink vs brown noise) to set a rule for interpreting an identical single-whisker deflection (lick in W+ and withhold in W− contexts) while always licking to a brief 10 kHz tone. Behaviorally, animals acquire the rule and switch rapidly at block transitions and take a few trials to fully integrate the context cue. What's nice about this behavior is the separate auditory cue, which shows the animals remain engaged in the task, so it's not just that the mice check out (i.e., become disengaged in the W- context). The authors then use optical tools, combining cortex-wide optogenetic inactivation (using localized inhibition in a grid-like fashion) with widefield calcium imaging to map what regions are necessary for the task and what the local and global dynamics are. Classic whisker sensorimotor nodes (wS1/wS2/wM/ALM) behave as expected with silencing reducing whisker-evoked licking. Retrosplenial cortex (RSC) emerges as a somewhat unexpected, context-specific node: silencing RSC (and tjS1) increases licking selectively in W−, arguing that these regions contribute to applying the "don't lick" policy in that context. I say somewhat because work from the Delamater group points to this possibility, albeit in a Pavlovian conditioning task and without neural data.

The widefield imaging shows that RSC is the earliest dorsal cortical area to show W+ vs W− divergence after the whisker stimulus, preceding whisker motor cortex, consistent with RSC injecting context into the sensorimotor flow. A "Context Off" control (continuous white noise; same block structure) impairs context discrimination, indicating the continuous background is actually used to set the rule (an important addition!) Pre-stimulus functional-connectivity analyses suggest that there is some activity correlation that maps to the context presumably due to the continuous background auditory context. Simultaneous opto+imaging projects perturbations into a low-dimensional subspace that separates lick vs no-lick trajectories in an interpretable way.

In my view, this is a clear, rigorous systems-level study that identifies an important role for RSC in context-dependent sensorimotor transformation, thereby expanding RSC's involvement beyond navigation/memory into active sensing and action selection. The behavioral paradigm is thoughtfully designed, the claims related to the imaging are well defended, and the causal mapping is strong.

Comments on revisions:

The authors have been responsive to the prior review and I think the manuscript is a valuable and important addition to the literature.

# Referee report: Reviewer #2 (Public review)

**DOI:** 10.7554/eLife.109717.3.sa2

Summary:

The authors aim to understand the neural basis of context-dependent sensory processing and decision-making.

Strengths:

They used an innovative behavioral paradigm where the action-outcome association changes independent of the sensory stimulus. This allowed the authors to disentangle the effect of behavioral context on sensory processing in RSC. Using this approach combined with optogenetic silencing, they discover that RSC activity is necessary for suppressing a lick response when the stimulus switches to the unrewarded context. The authors provide compelling evidence that the RSC is an important node of context-dependent sensory processing.

Weaknesses:

Sensory processing appears to be entangled with jaw/tongue movement initiation. Nonetheless, it is clear that RSC and motor cortex convey contextual signals with a very short latency.

Comments on revisions:

Thank you for updating the manuscript. Good work.

# Author comment: Author response

**DOI:** 10.7554/eLife.109717.3.sa3

**Authors:** Pol Bech, Robin Dard, Jules Lebert, Lana Smith, Axel Bisi, Anthony Renard, Sylvain Crochet, Carl CH Petersen

**Affiliations:** École Polytechnique Fédérale de Lausanne (EPFL) Lausanne Switzerland; École Polytechnique Fédérale de Lausanne Lausanne Switzerland; École Polytechnique Fédérale de Lausanne Lausanne Switzerland; École Polytechnique Fédérale de Lausanne Lausanne Switzerland; École Polytechnique Fédérale de Lausanne Lausanne Switzerland; École Polytechnique Fédérale de Lausanne Lausanne Switzerland; École Polytechnique Fédérale de Lausanne Lausanne Switzerland; École Polytechnique Fédérale de Lausanne Lausanne Switzerland

The following is the authors’ response to the original reviews.

**Public Reviews:**

**Reviewer #1 (Public review):**

Summary

The strength of this manuscript lies in the behavior: mice use a continuous auditory background (pink vs brown noise) to set a rule for interpreting an identical single-whisker deflection (lick in W+ and withhold in W− contexts) while always licking to a brief 10 kHz tone. Behaviorally, animals acquire the rule and switch rapidly at block transitions and take a few trials to fully integrate the context cue. What's nice about this behavior is the separate auditory cue, which shows the animals remain engaged in the task, so it's not just that the mice check out (i.e., become disengaged in the W- context). The authors then use optical tools, combining cortexwide optogenetic inactivation (using localized inhibition in a grid-like fashion) with widefield calcium imaging to map what regions are necessary for the task and what the local and global dynamics are. Classic whisker sensorimotor nodes (wS1/wS2/wM/ALM) behave as expected with silencing reducing whisker-evoked licking. Retrosplenial cortex (RSC) emerges as a somewhat unexpected, context-specific node: silencing RSC (and tjS1) increases licking selectively in W−, arguing that these regions contribute to applying the "don't lick" policy in that context. I say somewhat because work from the Delamater group points to this possibility, albeit in a Pavlovian conditioning task and without neural data. I would still recommend the authors of the current manuscript review that work to see whether there is a relevant framework or concept (Castiello, Zhang, Delamater, 'The retrosplenial cortex as a possible 'sensory integration' area: a neural network modeling approach of the differential outcomes effect of negative patterning', 2021, Neurobiology of Learning and Memory).

The widefield imaging shows that RSC is the earliest dorsal cortical area to show W+ vs W− divergence after the whisker stimulus, preceding whisker motor cortex, consistent with RSC injecting context into the sensorimotor flow. A "Context Off" control (continuous white noise; same block structure) impairs context discrimination, indicating the continuous background is actually used to set the rule (an important addition!) Pre-stimulus functional-connectivity analyses suggest that there is some activity correlation that maps to the context presumably due to the continuous background auditory context. Simultaneous opto+imaging projects perturbations into a low-dimensional subspace that separates lick vs no-lick trajectories in an interpretable way.

In my view, this is a clear, rigorous systems-level study that identifies an important role for RSC in context-dependent sensorimotor transformation, thereby expanding RSC's involvement beyond navigation/memory into active sensing and action selection. The behavioral paradigm is thoughtfully designed, the claims related to the imaging are well defended, and the causal mapping is strong. I have a few suggestions for clarity that may require a bit of data analysis. I also outline one key limitation that should be discussed, but is likely beyond the scope of this manuscript.

Major strengths

(1) The task is a major strength. It asks the animal to generate differential motor output to the same sensory stimulus, does so in a block-based manner, and the Context-Off condition convincingly shows that the continuous contextual cue is necessary. The auditory tone control ensures this is more than a 'motivational' context but is decision-related. In fact, the slightly higher bias to lick on the catch trials in the W+ context is further evidence for this.

(2) The dorsal-cortex optogenetic grid avoids a 'look-where-we-expect' approach and lets RSC fall out as a key node. The authors then follow this up with pharmacology and latency analyses to rule out simple motor confounds. Overall, this is rigorous and thoughtfully done.

(3) While the mesoscale imaging doesn't allow for cellular resolution, it allows for mapping of the flow of information. It places RSC early in the context-specific divergence after whisker onset, a valuable piece that complements prior work.

(4) The baseline (pre-stim) functional connectivity and the opto-perturbation projections into a task subspace increase the significance of the work by moving beyond local correlates.

Key limitation

The current optogenetic window begins ~10 ms before the sensory cue and extends 1s after, which is ideal for perturbing within-trial dynamics but cannot isolate whether RSC is required to maintain the context-specific rule during the baseline. Because context is continuously available, it makes me wonder whether RSC is the locus maintaining or, instead, gating the context signal. The paper's results are fully consistent with that possibility, but causality in the pre-stimulus window remains an open question. (As a pointer for future work, pre-stimulusonly inactivation, silencing around block switches, or context-omission probe trials (e.g., removing the background noise unexpectedly within a W+ or W- context block), could help separate 'holding' from 'gating' of the rule. But I'm not suggesting these are needed for this manuscript, but would be interesting for future studies.)

We thank the reviewer for the comprehensive summary of our work.

We also thank the reviewer for highlighting the work from the Delamater group (Castiello et al., 2021), and we now briefly discuss this paper on P. 14 Lines 434-437 writing: “RSC was shown to contribute to negative patterning in behavioral tasks requiring rats to learn that the simultaneous presentation of two stimuli lead to an opposite outcome than each individual stimulus (Castiello et al., 2021).”

We also agree with the reviewer’s noted ‘Key limitation’ regarding the role of RSC as either maintaining context representation or serving a gating function. The reviewer proposes an exciting set of further experiments inactivating RSC at different time points to investigate when RSC activity is needed. We hope to carry out such experiments in the future. We now include a brief discussion of this interesting point on P. 14-15 Lines 455-459 writing: “First, further inactivation experiments would shed light on the timing at which RSC activity is necessary for the integration of contextual information. Specifically, it would be of great interest to inactivate RSC at different time points such as during the intertrial interval or at the transition between contexts.”

We have of course also addressed each of the more detailed comments from the “Recommendations for the authors” section, please see below.

**Reviewer #2 (Public review):**

Summary:

The authors aim to understand the neural basis of context-dependent sensory processing and decision-making.

Strengths:

They used an innovative behavioral paradigm where the action-outcome association changes independent of the sensory stimulus. This theoretically allows the authors to disentangle the effect of behavioral context on sensory processing. Using this approach combined with optogenetic silencing, they discover that RSC activity is necessary for suppressing a lick response when the stimulus switches to the unrewarded context.

Weaknesses:

Sensory processing appears to be entangled with jaw/tongue movement initiation. Activity in M1 and RSC during auditory-evoked lick responses appears to be identical to activity during whisker-evoked lick responses, indicating that movement initiation is the main driver of M1/RSC activity, rather than changes in the flow of sensory information. If sensory information were the main driver of the initial M1/RSC response, then auditory evoked responses should have a longer latency. Perhaps this is beyond the resolution of the calcium indicator or imaging frame rate. It is not clear from the data shown if differences in S1 activity when comparing W+ and W- stimulation are caused by context-sensitive sensory processing or whisker movement following whisker deflection.

We thank the reviewer for the comments on our work and we agree that separating sensory processing and movement initiation is very important. In the revised manuscript, we have carried out several new analyses to specifically address the points of the reviewer. The most important point is that context-dependent activity in RSC emerges at ~50 ms after the whisker stimulus, which precedes any differences in movements of the jaw or whisker. Although sensory and motor representations become increasingly entangled after stimulus delivery, we think that the first ~100 ms after the whisker stimulus is a relatively safe period for analysing sensory processing and decision making before overt context-dependent differences in movements.

Addressing the specific point “Activity in M1 and RSC during auditory-evoked lick responses appears to be identical to activity during whisker-evoked lick responses, indicating that movement initiation is the main driver of M1/RSC activity, rather than changes in the flow of sensory information.” - We have now directly compared the pattern of cortical activity evoked by whisker and auditory stimuli in correct trials in the W+ context (new Figure 3 – figure supplement 2). As expected, activity in wS1/wS2 and A1 is stronger in whisker and auditory trials respectively, following their sensory modalities. However, we also evidence a stronger response of wM1/wM2 in whisker trials as early as 40 to 60 ms following the stimulus, showing the specificity to the whisker system. We also observe a stronger response of RSC to whisker than to auditory stimulus. The auditory and whisker evoked responses are therefore different.

Addressing the specific point “If sensory information were the main driver of the initial M1/RSC response, then auditory evoked responses should have a longer latency. Perhaps this is beyond the resolution of the calcium indicator or imaging frame rate.” – As stated above, the responses to auditory and whisker stimuli are different.

Addressing the specific point “It is not clear from the data shown if differences in S1 activity when comparing W+ and W- stimulation are caused by context-sensitive sensory processing or whisker movement following whisker deflection.” - We think that the data shown in Figure 3F-H indicate that differences in S1 activity when comparing W+ and W- stimulation are not directly caused by context-sensitive sensory processing. On P. 9 Lines 270273 we write: “Early after stimulus onset, whisker deflection evoked similar activation of primary and secondary whisker somatosensory cortices (wS1 and wS2) in both W+ and W− contexts.” Indeed, context separation in wS1/wS2 only emerged later than 100 ms, which is indeed confounded by the difference in movement evoked by the sensory stimulus (now quantified in new Figure 3 – figure supplement 4). On the contrary RSC and wM1/2 responses to the whisker stimulus were different in W+ and W- at early time points (~50 ms for RSC and ~80 ms for wM1/2) which is consistent with context dependent sensory processing. At least 2 hypotheses could explain the absence of early difference in whisker evoked activity in wS1/wS2 between W+ and W-. The first one is that sensory activity in wS1/wS2 is not modulated by contextual information at all, while the alternative option would imply that sensory activity is mediated by different neuronal populations depending on context with an overall similar average response. We think this is an interesting question which we hope to address in future experiments using *Neuropixels* recordings and multiphoton cellular imaging to address the single neuron representation of whisker stimulus in wS1/wS2 according to context in the task presented here.

We have of course also addressed each of the more detailed comments from the“Recommendations for the authors” section, please see below.

**Recommendations for the authors:**

**Reviewer #1 (Recommendations for the authors):**

Suggestions to strengthen the manuscript (no new data collection)

(1) The block-switch dynamics were clearly demonstrated behaviorally. It would be very powerful to mirror this with an analysis of neural data around the block-switch: how do the various areas adjust immediately after a shift in the continuous contextual sound? Does the RSC show any evidence of changing activity patterns? How does the within-trial activity dynamic look as a function of the number of trials from the context switch? This could be done with the data collected for Figure 3 (for within-trial dynamics), but also for the pre-stimulus baseline activity data (Figure 4A-B).

We thank the reviewer for raising this interesting point. We have now investigated the change of cortical activity at the transition between contexts (new Figure 3 – figure supplement 5). At the context transition, both to W+ and to W- contexts, we observed a rapid activation of the auditory cortex (new Figure 3 – figure supplement 5A). In addition, there appeared to be a slightly higher activation of RSC when transitioning to W- rather than to W+ (new Figure 3 – figure supplement 5A). In the future, it will be of great interest to further investigate this phenomenon.

We also evaluated the whisker deflection-evoked responses of the different cortical regions according to the number of whisker trials from context switch (new Figure 3 – figure supplement 5B&C). This analysis revealed that while the sensory response in wS1 and wS2 were constant over the time course of a context block, the response of wM1/2 and especially RSC became progressively lower in the W- context, consistent with the behavioral results in Figure 1 supporting time-dependent contextual integration.

Overall, these results strengthen the role of RSC and wM1/2 in integrating contextual information to guide the response to the whisker stimulus, and we thank the reviewer for raising this important point.

(2) It might be useful to state 'earliest among the imaged dorsal cortical areas,' and briefly acknowledge potential subcortical contributors (since those were not explored and could be earlier than cortical areas).

We agree with the reviewer. In the Summary, on P. 2 Line 39-40 we now write: “Widefield calcium imaging revealed that retrosplenial cortex was the first dorsal cortical area to show context discrimination in response to whisker stimulation”. On P. 8 Lines 257-258, we now write: “To investigate the spatiotemporal neural dynamics underlying task execution, we recorded calcium activity across the dorsal cortex in transgenic mice”. On P. 13 Lines 416-420 we now write: “Functional imaging of cortical activity with two different genetically-encoded calcium indicators each showed similar spatiotemporal dynamics of whisker sensory processing with the earliest contextdependent divergence in signalling being detected in RSC, out of the imaged dorsal cortical areas (Figure 3).” On P. 15 Lines 470-473, we now write: “Finally, it is of course important to note that many subcortical regions (as well as non-dorsal cortical regions, which were not imaged) are likely to contribute importantly to context-dependent task performance.”

(3) Fit a simple exponential/logistic to lick probability vs time-since-switch (your Figure 1Hstyle analysis) to report a time constant with CIs; it will help quantify the integration of the continuous cue.

We thank the reviewer for this suggestion. We have fitted an exponential to the grand average data to quantify the time constants for integration of contextual information before the presentation of the first whisker stimulus of the block (see new Figure 1H). On P. 6 Lines 170-173 we now write: “To assess whether this temporal integration would differ between contexts we fitted an exponential to the time evolution of the lick probability. This suggested a faster transition to the W+ context than to the W- context (W+ time constant: 9.4 s, W- time constant: 15.5 s) (Figure 1H).”

(4) Because catch-trial false alarms are higher in W+ than W−, report per-context d′ and criterion for whisker trials (using signal detection theory); this separates sensitivity from bias and makes the behavioral shift more interpretable. It is also further proof that the behavior is contextual (versus a compound stimulus, for example).

We have computed the d’ and criterion for the whisker trials in the W- and W+ contexts. (see new Figure 1 - figure supplementary 1D). As suggested by the reviewer, this further supports that the behavior is driven by contextual information.

(5) For the pre-stimulus seed-correlation analysis, can you regress out the pupil/jaw/whisker activity to confirm whether the context modulation is (or is not) movement-driven? It would be helpful to better understand whether the baseline correlation is driven by differences in lowlevel factors between the contexts, versus the higher-level decision rule/context.

The reviewer raises an interesting point. However, we did not find a straightforward way to regress out movements, and thus we leave this point for future in-depth analysis. On P. 11 Lines 354-357 we now write: “It is important to note that these context-dependent changes in resting-state functional connectivity could relate to the overt context-dependent movements in the prestimulus baseline (Figure 1I&J) and/or a manifestation of higher-level internal rule representations.”

(6) For the earliest divergence analysis, is this consistent across animals and across sessions within animals? Can you show per-mouse distributions of first-crossing times (d′>2) for RSC vs wM1/2/wS2? This would help provide confidence in this key finding.

The d’ presented in Figure 3H is computed as the discriminability between contexts at the population level, meaning that at each timepoint (from Figure 3F) we compared the 2 distributions built on N=6 mice. As such if the divergence between context was not consistent across animals this d’ would be low. That said, as suggested by the reviewer, we further investigated this context divergence at single mouse level and single session level. Our analysis supporting the main finding (Figure 3F-H) is shown in new Figure 3 – figure supplement 3.

First, we show the results for a single mouse across sessions in Figure 3 – figure supplement 3A. We show the stimulus aligned activity in correct whisker trials in both contexts for the 3 recording sessions. For each session we quantified the main effect size defined as the difference of the trial average between contexts. Plotting the difference of mean response, we consistently observed that RSC ramps-up before wM1/2 for the 3 sessions.

Second, across all individual mice: we further aggregated the session average responses to show discriminability between context for each region at the single mouse level (Figure 3 – figure supplement 3B). We show that RSC is the first region to exhibit context separation in 4 out of the 6 mice that we recorded. In 2 other mice all regions seemed to show context separation but without clear temporal ordering.

Finally, when averaging across mice, we observed a clear separation and first discrimination in RSC (Figure 3F-H and Figure 3 – figure supplement 3C).

Overall, these further analyses suggest that the early divergence of RSC activity appears to be robust with a consistent mean difference in single sessions and single mice, as well as across the population of mice. We think this analysis has strengthened our manuscript and we thank the reviewer for the valuable suggestion.

(7) For the opto mapping data, could you provide P(lick) effect sizes with CIs per grid site? It would also be nice to summarize the qualitative dichotomy: RSC/tjS1 increases licking in W−; canonical wS1/wS2/wM/ALM decreases licking across contexts (to my understanding).

We now provide the P(lick) effect sizes for the main cortical areas studied in the paper in Figure 2 – figure supplement 1C. This shows the relative change in lick probability in optogenetic trials compare to control trials for each mouse.

**Reviewer #2 (Recommendations for the authors):**

(1) Do mice move their whiskers after stimulus onset? If so, are these movements dependent on behavioral context? What causes the increase in S1 activity during auditory-evoked response trials?

To answer the reviewer’s questions we have further investigated whisker movements following the sensory stimuli (whisker and auditory correct trials) in both contexts. The results of this analysis are presented in new Figure 3 – figure supplement 4.

We find that mice move their whiskers shortly after the whisker stimulus in both contexts. The time course of whisker angle in correct whisker trials is similar in both contexts with a discriminability index (d’) consistently below 1. The whisker speed in response to stimulus is slightly higher in the W+ context compared to W- with a d’ slightly above 1 after ~100 ms. We also observed evoked whisker movements in auditory trials independent of context. Thus, whisker movements are indeed evoked by the sensory stimuli, but the overall context-dependent modulation of whisker movements is weak. The early differences in whisker-evoked cortical activity in W+ compared to W- contexts are therefore more likely related to the integration of contextual information than to differences in evoked movements.

The reviewer is correct to point out that wS1 activity increases in auditory trials (Figure 3E). The response is initially very weak, but becomes more prominent after ~100 ms following the auditory tone. We do not know the underlying mechanisms, but there are several likely explanations. First, as discussed above, there are indeed some whisker movements evoked in response to the auditory stimulus (Figure 3 – figure supplement 4), which could result in sensory input to wS1. Equally, the increase could relate to licking, given the broad representation of movements in cortex and an appropriate reaction time in auditory trials (Figure 3C). Alternatively, wS1 activity in auditory trials could also be related to input connectivity from auditory cortex, top-down input from frontal cortex or subcortical regions such as high-order POm.

(2) What do the authors think is causing the W+ vs W- difference in S1/S2 activity approximately 100ms after whisker deflection?

The late W+ vs W- difference in wS1/wS2 activity could be explained by several factors. First this could be due to the difference in whisker movements after ~100 ms as shown in Figure 3 – figure supplement 4. Second this could be driven by the lick vs no lick activity (see reaction time in Figure 3C for whisker trials ~110 ms). Finally, this could be partly due to some movement independent top-down contextual information reaching wS1/wS2 at late time points. Overall, our claim in the paper is that there was no contextual difference in whisker primary and secondary cortices at early time points (before movement). On P. 9 Lines 270-273 we explicitly write: “Early after stimulus onset, whisker deflection evoked similar activation of primary and secondary whisker somatosensory cortices (wS1 and wS2) in both W+ and W− contexts.” In contrast, our main findings are grounded in the divergence of cortical activity in RSC and wM1/2 at early time points (<100 ms).

(3) The choice of PC3 seems arbitrary. Is there no task-relevant information in PC1 and PC2?

We appreciate the point raised by the reviewer and have clarified the reasoning leading to PC3 selection in the main text, where on P. 12-13 Lines 384-391 we now write: “The loadings of the first principal components were uniformly distributed and could reflect a late movement driven activation distributed across all cortical areas (Figure 4 – figure supplement 2C&D). PC2 loadings show variation along the anteroposterior axis that could reflect differences between sensory and motor regions but its time course does not separate between lick and no lick in control conditions (Figure 4 – figure supplement 2C&D). The loadings of PC3 highlighted task-related cortical regions and its time course exhibited clear differences comparing lick and no-lick trials.” In addition, we now also show the time courses for PC1 and PC2 in Figure 4 – figure supplementary 2D.

Overall, the reasoning is the following:

PC1 has spatially-homogeneous positive loadings (Figure 4 – figure supplementary 2C) and activity along PC1 gradually ramps up following sensory stimulation (Figure 4 – figure supplementary 2D). It is likely driven by widespread activation of the cortex following the whisker stimulus and the lick response. As such we believe that the taskrelated information captured by PC1 is movement related and not necessarily informative about processing of whisker and context.

PC 2 has loadings varying along the antero-posterior axis (Figure 4 – figure supplementary 2C), which could be relevant for the task, but its time-course does not discriminate between lick and no lick neither in W+ nor W- (Figure 4 – figure supplementary 2D).

PC3 has both loadings that vary between several cortical regions involved in the task (Figure 4 – figure supplementary 2C) and a time course that separates between lick and no lick in both contexts (Figure 4 – figure supplementary 2D). We thus focus on PC3 to investigate the effect of optogenetic inactivation on whisker stimulus evoked activity.

The remaining components beyond PC3 contain a very small fraction of variance and were thus not considered.

(4) Figure 3 - Supplement 1: What explains the change in fluorescence in GFP/tdT mice during W+ stimulation? Is it brain movement on the z-dimension? Could this explain differences in calcium imaging results?

We thank the reviewer for this question. The nature of intrinsic signals is a complex topic, but brain movement is unlikely to contribute importantly, because under similar behavioral conditions we (and others) typically find brain movements to be on the scale of a few microns. The three most widely-reported contributions to intrinsic optical changes in cortex relate to:

(i) Light scattering – as neurons integrate synaptic inputs and fire action potentials, the neuronal elements swell slightly due to the ionic and water fluxes (see for example Vincis et al. Cell Reports 2015, doi: 10.1016/j.celrep.2015.06.016). This reduces the refractive index mismatch between the intracellular and extracellular space. This in turn reduces light scattering, which could result in fluorescence increases.

(ii) Hemodynamics – changes in blood volume and changes in oxygenation/deoxygenation will change the absorption of light at different wavelengths, in an activity-dependent manner (also forming the basis of BOLD fMRI signals).

(iii) Flavoproteins – endogenous fluorescent proteins, such as flavoproteins present at high levels in mitochondria, have been reported to change their fluorescence depending upon neuronal activity, presumably in relationship to increased mitochondrial activity.

We therefore think it is very important to image GFP/tdTomato-expressing mice as controls, and we would suggest that this should be carried out more commonly in the field. Indeed, similar to our results, another study (Yogesh et al., eLife 2025, doi: 10.7554/eLife.104914) recently reported upon the importance of carefully examining intrinsic fluorescence changes, which were found to be present in both wide-field and two-photon imaging of GFP expressing mice.

Our results reported in Figure 3 – figure supplement 1, show that GFP/tdTomato signals over the first ~120 ms following whisker stimulation were much smaller that the equivalent changes in GCaMP6f/jRGECO1a-expressing mice, and therefore would only have a minor contribution to our analyses. However, we refrained from analysing fluorescence changes at later post-stimulus times, because the intrinsic signals indeed become increasingly prominent as the mice initiate licking.
